# Interactive Effects of Nitrogen and Potassium on Grain Yield and Quality of Waxy Maize

**DOI:** 10.3390/plants11192528

**Published:** 2022-09-27

**Authors:** Qijian Zhang, Guanghao Li, Weiping Lu, Dalei Lu

**Affiliations:** Jiangsu Key Laboratory of Crop Genetics and Physiology/Jiangsu Key Laboratory of Crop Cultivation and Physiology/Jiangsu Co-Innovation Center for Modern Production Technology of Grain Crops, Yangzhou University, Yangzhou 225009, China

**Keywords:** waxy maize, interactive effects of N and K, yield, pasting and thermal property

## Abstract

Reasonable fertilization of nitrogen (N) and potassium (K) had significant effects on maize growth. In this experiment, two N levels (N180 and N225 kg ha^−1^) and four K treatments (K0, K75, K150 and K75 + 75 kg ha^−1^) were set to study the effects of combined application of N and K on the grain yield and quality of waxy maize. The results showed that grain yield increased with increasing K under the same N level, and top-dressing K further increased the grain yield. K application increased starch content significantly at N180 and decreased significantly at N225, while the protein content increased significantly at the two N levels. The grain starch content with the K75 + 75 treatment increased by 5.8% and 9.0% compared with K150 at the two N levels, and the protein content decreased by 2.9% and 4.7%. Application of K increased the retrogradation enthalpy (Δ*H*_ret_) and retrogradation percentage (%*R*) at N180. At N225, the Δ*H*_ret_ and %*R* of K75 and K150 decreased, while those of K75 + 75 increased. The Δ*H*_ret_ and %*R* under K75 + 75 at N180 were lower than N225. Under these experiment conditions, 75 kg ha^−1^ K_2_O at sowing date and top-dressed 75 kg ha^−1^ K_2_O at jointing stage (V6) under the conditions of appropriate N reduction could not only effectively improve the pasting and thermal properties of waxy maize flour, but also stabilized the grain yield.

## 1. Introduction

Waxy maize is a special crop, characterized by a unique structure of starch, which creates enormous possibilities for its application [1]. Among the various types, waxy maize starch is distinct, with starch composed of nearly pure amylopectin, which provides advantages of viscosity, stability, and retrogradation [2]. Waxy maize was originally used mainly for industrial production, and only to a small extent for food production [3,4]. With the enlargement of waxy maize purposes, it has become an important raw material for food industries, textiles, paper-making and feedstuff [5].

Nitrogen (N) and potassium (K) are essential nutrients for crop growth and development. N participates in plant physiological processes as important components such as enzymes, hormones and amino acids [6]. K is beneficial to promote carbohydrate and N metabolism, and K ions in the form of compensation charge significantly promote the absorption and utilization of N and phosphorus (P) in crops, thereby improving the quality of crop products [7]. Appropriate N was beneficial to starch accumulation and protein synthesis in maize grains [8], and affected the size and morphology of starch [9]; thus, it changed the pasting properties of maize starch and flour [10,11]. Appropriate K was beneficial to the improvement of maize yield, protein content and starch accumulation [12]. The balanced application of N, P and K fertilizers could optimize the pasting and thermal property of waxy maize flour, with higher peak viscosity (PV), trough viscosity (TV), final viscosity (FV), breakdown viscosity (BD), gelatinization enthalpy (Δ*H*_gel_), Δ*H*_ret_ and %*R* [13,14]. N fertilizer had a dose-effect on rice grain quality: an appropriate N increased the size of starch granules and improved the pasting property, but excessive N deteriorated the grain quality [15]. K fertilizer increased rice gel consistency and grain protein content, but simultaneous application of N and K had no significant effects on gel consistency [16]. Additional K under the premise of reasonable N could effectively improve the grain yield and protein content in rice [17]. The suitable N, P and K fertilizer increased the pasting onset temperature (*T*_o_), FV and setback viscosity (SB) of rice flour, but decreased the PV and BD [18]. Studies on wheat also showed that both N and K fertilizers increased the grain yield and protein content [19]. Excessive N application led to a large accumulation of protein and small starch granules, resulting in a decrease in starch viscosity [20]. Appropriate N application increased the PV and FV of wheat flour [21]. K enhanced the supply of sucrose during grain filling, and increased the activity of grain sucrose synthase, which was beneficial to the decomposition of sucrose in grains and produced sufficient precursors for starch synthesis. In addition, N and K also increased the soluble starch synthase and starch granule bound starch synthase activities in grains [22].

Since the 20th century, breeding and industry of waxy maize has developed rapidly in China, and its planting area has exceeded 800,000 ha [23], which has undoubtedly increased the amount of fertilizer used. Although growth, grain yield and quality of maize depend upon substantial N inputs, finding the optimum concentration and avoiding over and under-use of N fertilizer is very important to maintain maximum productivity with reduced costs, resource waste and environmental pollution [24]. In recent years, the excessive application of N fertilizer has caused environmental pollution [25], and the lack of K has limited the yield and quality improvement of maize grain [26]. It is an inevitable trend to apply reasonable and balanced fertilization according to local conditions. Previous studies on the effects of N–K interactions on crops mainly focused on yield and the physiological mechanisms affecting yield formation [27,28], but the effects on grain yield and quality of waxy maize were rarely reported.

Therefore, the objectives of this study were: (1) to investigate the interactive effects of N and K on grain component content, yield and its components of waxy maize; (2) to investigate the interactive effects of N and K on the pasting property and thermal property of waxy maize flour; and (3) to determine the appropriate application of N and K fertilizer in the production of spring-sown waxy maize in Southern China.

## 2. Results

### 2.1. Grain Yield and Yield Components

There were significant differences in kernel number of ears, 1000-kernel weight and grain yield of waxy maize between K, year (Y), and the interaction (K × Y). N, K and Y have significant effects on grain yield, and Y has the largest effect. The interaction (N × K) and the interaction (N × K × Y) have little or no significant effect on grain yield (Table 1). According to Table S1, compared with K0, K fertilizer significantly increased the number of ears, 1000-kernel weight and grain yield at the two N levels. Average grain yield in 2021 was higher than 2020, and SYN11 had higher grain yield than SYN5. At N180, kernel number of K75, K150 and K75 + 75 increased by 5.97%, 8.33% and 9.23%, 1000-kernel weight increased by 2.69%, 2.91% and 3.80%, and grain yield increased by 8.58%, 11.44% and 13.35% compared with K0. At N225, kernel number of K75, K150 and K75 + 75 increased by 9.33%, 8.26% and 8.08%, 1000-kernel weight increased by 1.17%, 4.33% and 6.46%, and grain yield increased by 10.66%, 12.71% and 15.10%.

The trends of 1000-kernel weight and grain yield were identical at the two N levels, and showed K75 + 75 > K150 > K75 > K0. At the same K level, 1000-kernel weight and grain yield in N180 were significantly lower than in N225. N225K75 and N180K150 significantly increased the kernel number of ears and grain yield with additional N or K fertilizer compared with N180K75. N225K150 significantly increased the 1000-kernel weight and grain yield with additional N and K fertilizer compared with N180K75. Compared with K150, the kernel number of ears, 1000-kernel weight and grain yield were further increased by K75 + 75, but the difference in kernel number of ears was not significant.

### 2.2. Grain Component Content

There were significant differences in starch and soluble sugar contents between N, K, Y, the interaction (N × K), the interaction (K × Y) and the interaction (N × K × Y), while K, the interaction (N × K), the interaction (N × Y), the interaction (K × Y) and the interaction (N × K × Y) have significant effects on protein content (Table 2). N had no significant effect on protein content, which could be due to the small difference between the two N levels.

According to Table S2, starch content in 2020 was lower than 2021, and soluble sugar and protein contents in 2020 were higher than 2021. Starch and soluble sugar contents in SYN5 were higher than SYN11, and protein contents in SYN5 were lower than SYN11. Compared with K0, starch content was significantly increased in K75, K150 and K75 + 75 at the N180 level, soluble sugar content was decreased in K75 and increased in K150 and K75 + 75, and protein content was increased in K75 and K150 and decreased in K75 + 75. At N225, application K decreased the contents of starch and soluble sugar and increased protein content under K75 and K150, while the starch content of K75 + 75 was higher than K0, and soluble sugar content was lower than K0.

Compared with N180K75, the starch and protein content of N225K75 and N180K150 significantly decreased and soluble sugar content increased. Compared with N180K75, the starch content of N225K150 significantly decreased and soluble sugar content increased, and there was no significant difference in protein content. Under the two N levels, starch content of K75 + 75 increased by 5.8% and 9.0%, and protein content decreased by 2.9% and 4.7% compared with K150.

### 2.3. Starch Granule Size and Distribution

**Figure 1:**
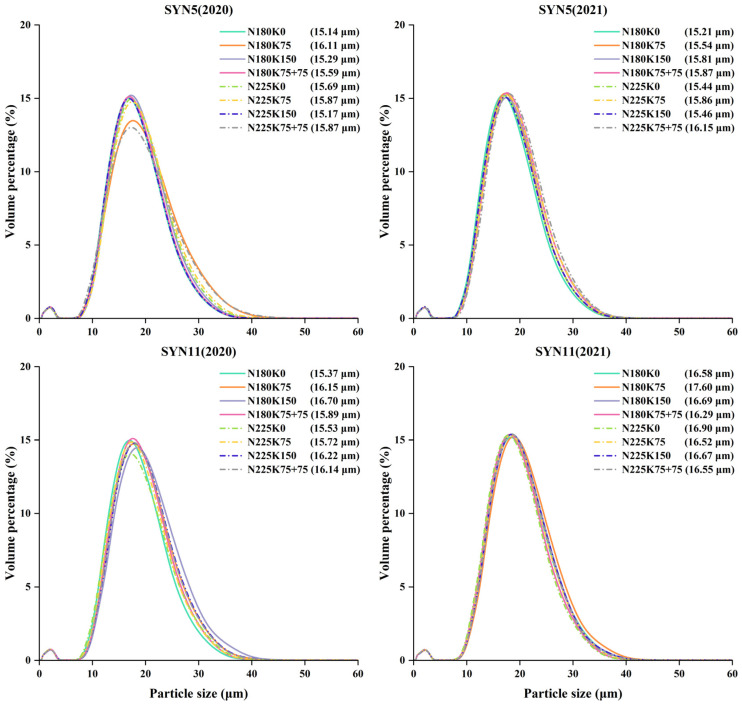
The volume distributions of waxy maize starch granules under different fertilization treatments. N180, N225: application 180 and 225 kg N ha^−1^; K0, K75 and K150: application 0, 75 and 150 kg K_2_O ha^−1^ at sowing date; K75 + 75: application 75 kg K_2_O ha^−1^ at sowing date and jointing stage (V6), respectively. Data in the bracket are the mean particle size. The figure shows the mean values of three replicates of the same treatment.

The size distribution of starch granules showed a typical bimodal pattern, and the variation of the effect of different N–K interactions on starch size distribution in 2020 was sharper than that in 2021 (Figure 1), which might be related to the higher rainfall during the grain filling period of waxy maize in 2020. Starch grain volume distribution and volume mean grain size differed significantly between years, varieties and fertilization treatments (Table S3). At the two N levels, K fertilization reduced the proportion of small starch grains (<5 μm) and increased the proportion of large starch grains (>15 μm) compared with K0, thus significantly increasing the mean grain size. N225K75, N180K150 and N225K150 significantly increased the proportion of small starch granules and decreased the volumetric mean particle size compared with N180K75 by adding N or K fertilizer or by adding N and K fertilizer together. At the same level of K, the proportion of small starch granules in K75 and K150 was significantly lower than that in N225, and the volumetric mean grain size was significantly higher in N180 than that in N225, but in K75 + 75, the proportion of small starch granules in N180 was significantly higher than that in N225, and the volumetric mean grain size in N180 was significantly lower than that in N225.

### 2.4. Pasting Properties of Waxy Maize Flour

**Figure 2:**
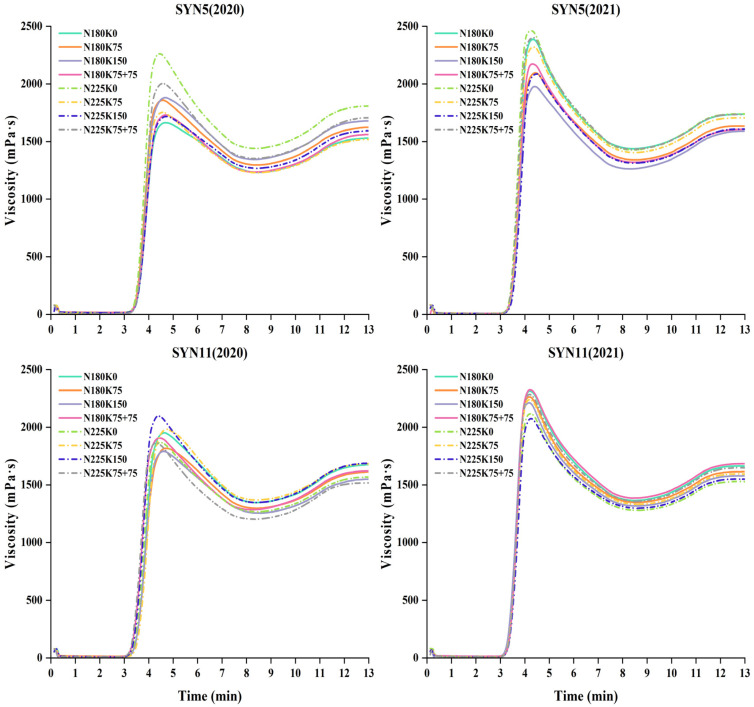
Pasting properties of waxy maize under different fertilization treatments. N180, N225: application 180 and 225 kg N ha^−1^; K0, K75, K150: application 0, 75 and 150 kg K_2_O ha^−1^ at sowing date; K75 + 75: application 75 kg K_2_O ha^−1^ at sowing date and jointing stage (V6), respectively. The figure shows the mean values of three replicates of the same treatment.

Interactions of N and K had significant effects on the flour pasting properties of waxy maize (Figure 2). PV between varieties and pasting temperature (*P*_temp_) between fertilization treatments were less affected by N–K interactions (Table S4). The PV, TV, BD and FV in 2020 were lower than those in 2021, but the SB and *P*_temp_ were higher than those in 2021. At the two N levels, the pasting properties of waxy maize flour in the combined application of N and K showed the same trend, with PV, TV, BD and FV all showing K0 > K75 + 75 > K75 > K150, and the SB all showing K75 + 75 > K0 > K150 > K75 (but the differences between K0 and K75 + 75 treatments were not significant, and between K75 and K150 treatments were not significant). At the same K level, the pasting viscosity under N180 was lower than N225.

Compared with N180K75, N225K75 increased the PV, TV, BD and FV of waxy maize flour and decreased the SB with additional N fertilizer; N180K150 decreased the PV, TV, BD and FV, and increased the SB with additional K fertilizer; N225K150 decreased PV, TV, BD and FV, and increased SB with additional N and K fertilizer.

### 2.5. Thermal Properties of Waxy Maize Flour

According to Table 3, there were significant differences in Δ*H*_gel_, and gelatinization temperature (*T*_o_, *T*_p_ and *T*_c_) between K treatments, no significant differences in Δ*H*_ret_ between N treatments, and significant differences in %R between the interaction (N × K). Overall, the Δ*H*_gel_ and gelatinization temperature (*T*_o_, *T*_p_ and *T*_c_) in 2020 were higher than those in 2021, but the Δ*H*_ret_ and %*R* were lower. At N180 level, the Δ*H*_gel_ decreased in K75 and K150 compared with K0, and the differences between treatments of *T*_o_, *T*_p_ and *T*_c_ were not significant, while the Δ*H*_ret_ and %*R* were increased. At the N225 level, compared with K0, the Δ*H*_gel_, *T*_o_ and *T*_p_ were increased in K75, and the Δ*H*_ret_ and %*R* were decreased; in the K150 treatment, the Δ*H*_gel_, Δ*H*_ret_ and %*R* was decreased, while the *T*_o_ and *T*_p_ were increased.

Compared with N180K75, the Δ*H*_gel_ of N225K75 and N180K150 treatments increased, and the Δ*H*_ret_ and %*R* decreased. Compared with N180K75, Δ*H*_gel_ of N225K150 increased, and Δ*H*_ret_ and %*R* significantly decreased. In comparison with N180K150, N180K75 + 75 showed significantly lower Δ*H*_gel_, and higher Δ*H*_ret_ and %*R*. In comparison with N225K150, Δ*H*_gel_, Δ*H*_ret_ and %*R* of N225K75 + 75 significantly increased.

### 2.6. Correlation Analysis

**Figure 3:**
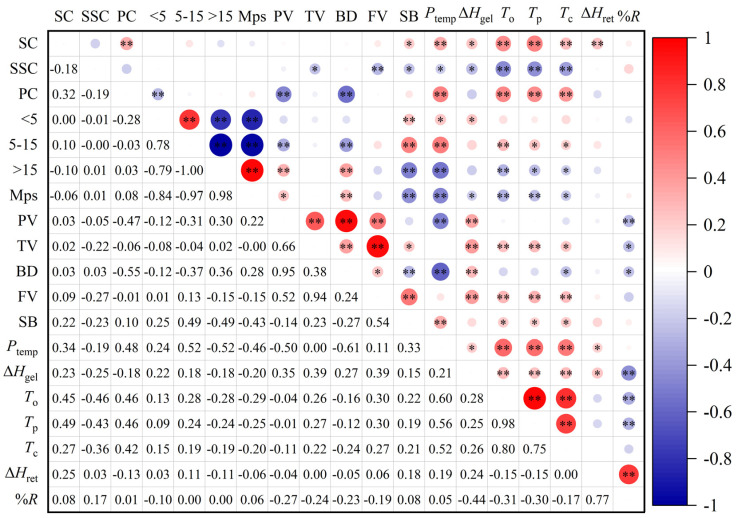
Correlation coefficients of grain quality parameters. SC: starch content; SSC: soluble sugar content; PC: protein content; <5: volume percentage of starch less than 5 μm; 5–15: volume percentage of starch between 5–15 μm; >15: volume percentage of starch more than 15 μm; Mps: mean particle size; PV: peak viscosity; TV: trough viscosity; BD: breakdown viscosity; FV: final viscosity; SB: setback viscosity; *P*_temp_: pasting temperature; Δ*H*_gel_: gelatinization enthalpy; *T*_o_: onset temperature; *T*_p_: peak gelatinization temperature; *T*_c_: conclusion temperature; Δ*H*_ret_: retrogradation enthalpy; %*R*: retrogradation percentage. * and ** represent significance at the 0.05 and 0.01 probability levels.

Correlation analysis (Figure 3) showed that there was a positive correlation between starch content and protein content, SB, *P*_temp_, Δ*H*_gel_, gelatinization temperature (*T*_o_, *T*_p_, *T*_c_) and Δ*H*_ret_. There was a negative correlation between soluble sugar content, SB, *P*_temp_, Δ*H*_gel_, and gelatinization temperature. Protein content was negatively correlated with PV and BD, and positively correlated with *P*_temp_ and gelatinization temperature. The starch volume percentage of particle size > 15 μm has a significant positive correlation with the mean particle size. The PV, TV, BD and FV have a significant positive correlation between pairwise comparisons. The gelatinization temperature had a significant positive correlation between pairwise comparisons.

## 3. Discussion

Previous studies have shown that reasonable N, P and K fertilization could significantly improve crop yield [29]. In terms of yield stability, the combined use of different fertilizers can be more effective than the application of single fertilizer alone [30]. The effect of N and K fertilization on crop yield was not a simple additive effect, but a complex interactive effect that promoted and constrained each other, and higher N and K fertilization might not necessarily increase yield, but may result in yield reduction [28]. The results of this experiment showed that combined application of N and K fertilizers significantly increased the grain yield of waxy maize compared with no K fertilizer. Compared with the basal application of K fertilizer, top-dressing K fertilizer at the jointing stage (V6) increased the grain yield, which was mainly influenced by the 1000-kernel weight and less affected by the kernel number. Many researchers have reported that maize yield increased in response to K fertilization [31,32]. However, there were also reports that K fertilizer had no effects on improving grain yield, although they reported increased K tissue concentrations as a result of K fertilization [33]. This may be influenced by various factors including soil, year, variety and climate.

Reasonable fertilization is one of the most effective measures to improve maize quality [34]. Compared with low N application, moderate N application increased the degradation and utilization of sucrose and starch synthesis, and accelerated the formation of maize starch granules [35]. The synthesis of protein and starch in the crop also required K; it facilitated carbohydrate metabolism and enzyme activation [36]. Reasonable N, P and K fertilization could increase starch and protein content in grains [37]. A long-term (1982 to 2000) field experiment showed that a greater grain protein content was produced using a balanced N–P–K fertilizer compared to using a fertilizer composed of only N and P in China [38]. The results of this experiment showed that the effect of N–K interactions on starch, soluble sugar and protein content of waxy maize was complex; compared with K0, the starch content of K75 and K150 treatments decreased, and the protein content increased with the increase of K fertilizer application under the two N levels. There was a similar phenomenon observed in a related study on wheat [22]. The co-application of N and K fertilizers led to higher grain protein content stability than the individually applied fertilizers [39]. By top-dressing K, the K75 + 75 treatment significantly increased the starch content and decreased the protein content under the two N levels. Previous research has shown that the application of K fertilizer at a ratio of 1:1 between sowing date and V6 of sweet-waxy maize significantly increased the starch content, which was mainly due to the increase of sucrose decomposition capacity by K fertilizer and accelerated starch synthesis [40].

Starch granule size and distribution were influenced by years, growing areas and environment [41]. In this study, we found that the regularity of starch granule size distribution differed between the two years, and the variation was larger in 2020. We also found that the effect of combined N and K fertilization on starch granule size was similar at the two N levels. Compared with K0, the proportion of small starch granules was reduced, and the proportion of large starch granules was increased under the combined N and K fertilization treatments, thus increasing the mean particle size of starch. Previous studies have shown that the PV of waxy maize starch was increased by a suitable amount of N fertilizer [6]. This study also showed that the PV, TV, BD and FV of waxy maize flour with combined application of N and K fertilizer at the two N levels all revealed a decreasing trend, and the decrease rate increased with the increase of K fertilizer; however, these parameters were increased in the treatment with additional K fertilizer at V6. The correlation analysis showed that protein content was significantly negatively correlated with PV and BD, indicating that high protein content was beneficial to the thermal stability of waxy maize flour viscosity. This may be due to the fact that protein contributed to limiting the expansion of starch granules during the pasting process of flour, making the expanded granules less susceptible to damage during the shearing process [42]. The ANOVA results showed that there were significant effects of year, variety and fertilization treatment on Δ*H*_gel_, and significant effects of year and treatment on Δ*H*_ret_ and %*R*. In most situations, combination of N and K fertilizers reduced the Δ*H*_gel_ and increased the Δ*H*_ret_ and %*R*. Under the two N levels, the application of K fertilizer with K75 + 75 increased the Δ*H*_ret_ and %*R* of waxy maize flour compared with K150, indicating that K supplementation at V6 promoted the regeneration of waxy maize flour. Previous studies in wheat have reported that larger starch granules inhibited the gelatinization of starch, thereby increasing the gelatinization temperature [43]. Our results showed that the mean starch granule size increased under different N–K interactions, but the gelatinization temperature did not show a significant difference.

## 4. Materials and Methods

### 4.1. Experimental Design

The field experiment was conducted at Jiangxinsha farm (31°48′ N, 121°53′ E) in Nantong City, Jiangsu Province, China, in 2020 and 2021. Suyunuo5 (SYN5) and Suyunuo11 (SYN11) were used as experimental materials. SYN5 is the control waxy maize variety of the regional test in southern China. SYN11 is widely planted in Jiangsu Province. The tested soil was sandy loam, and the soil nutrient content prior to sowing was as follows: average contents of organic matter, total N, alkali hydrolysable N, available P, and exchangeable K in the plow layer (0–20 cm) were 10.05 g kg^−1^, 0.86 g kg^−1^, 66.3 mg kg^−1^, 6.98 mg kg^−1^ and 67.25 mg kg^−1^ in 2020, and 13.08 g kg^−1^, 1.01 g kg^−1^, 58.97 mg kg^−1^, 6.06 mg kg^−1^ and 60.49 mg kg^−1^ in 2021.

A split-plot randomized complete block design (RCBD) was used, with two N fertilizer levels (main plots) and four K fertilizer treatments (sub-plots). The two N levels were 180 (N180) and 225 kg N ha^−1^ (N225). The four K treatments were 0 (K0), 75 (K75), 150 (K150) and 75 + 75 kg K_2_O ha^−1^ (K75 + 75). All treatments had a basal application of 75 kg N ha^−1^ and 75 kg P_2_O_5_ ha^−1^ at sowing date. N180 and N225 treatments were top-dressed 105 and 150 kg N ha^−1^ at V6. K75 and K150 treatments were applied 75 and 150 kg K_2_O ha^−1^ at sowing date. K75 + 75 treatment was applied 75 kg K_2_O ha^−1^ at sowing date and V6, respectively. Urea (46% N), superphosphate (12% P_2_O_5_) and potassium chloride (60% K_2_O) were used as the N, P and K fertilizer sources.

**Figure 4:**
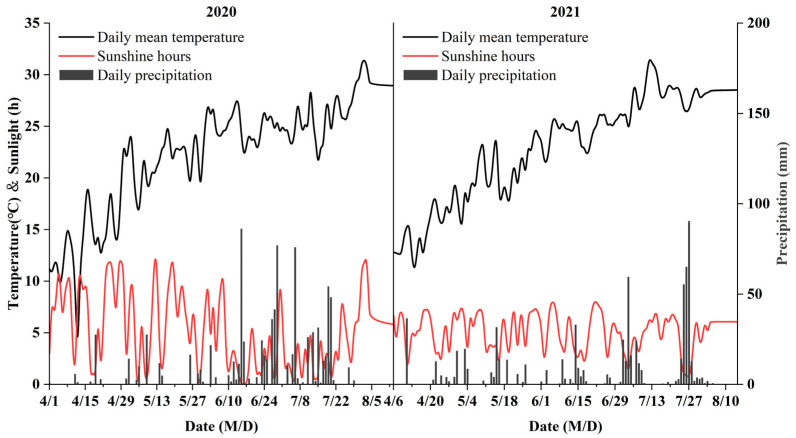
Daily precipitation, mean temperature and sunshine hours during maize growth seasons in 2020 and 2021.

Each plot was 43.2 m^2^ (3.6 m × 12.0 m), and the planting density was 60,000 plant ha^−1^ with double row planting (wide and narrow rows spacing were 0.8 and 0.4 m, respectively). The maize was sown on April 2 and harvested on August 5 in 2020, with a total growth period of 126 days. In 2021, it was sown on April 6 and harvested on August 8, with a total growth period of 125 days. The meteorological conditions of the field experiment were shown in Figure 4. The total rainfall and average temperature were 730.2 mm, 22.3 °C and 922.1 mm, 22.1 °C, in the maize-growing seasons.

### 4.2. Grain Yield and Yield Components

At maturity stages (R6), 30 ears were harvested from the middle row of each treatment using a continuous sampling method to determine the yield (standard water content of 14%) and its components.

### 4.3. Grain Component Content

Grain starch and soluble sugar content were measured by the anthrone–sulfuric acid method [44]; grain N content was determined by the Kjeldahl method following the procedure of AACC 46–10.01 [45]. Protein content was calculated as N content × 6.25.

### 4.4. Preparation of Starch and Flour Samples

Three uniform growth ears were selected for each treatment, and the stripped grains were mixed thoroughly. Then, 200 g of grains were randomly selected for the preparation of starch and flour samples. The grains (100 g) were steeped in 500 mL of 1 g/L NaHSO_3_ solution for 48 h at room temperature, and the starch was isolated following the method described in previous study [46]. The grains (100 g) were dried at 40 °C for 5 days, then ground with a grinder (FW100, Taisite, China) and passed through a 100 mesh sieve (0.149 mm) to determine the pasting and thermal property of maize flour.

### 4.5. Starch Granule Size and Distribution

The starch granule size and distribution were determined using a laser diffraction particle size analyzer (Mastersizer 2000, Malvern, England) with anhydrous ethanol as the dispersion medium, following the previously reported method of Yang et al. [47]. The mean particle size was defined as a volume-weighted average.

### 4.6. Pasting Property and Thermal Property of Waxy Maize Flour

According to the method of Yang et al. [47], the pasting and thermal properties of maize flour were analyzed. The pasting properties were determined using a rapid viscosity analyzer (RVA-TecMaster) from Newport Scientific (Australia) and analyzed with TCW3 (Thermo Cline for Windows) software. A sample suspension (28 g total weight; 10%, *w*/*w*, dry basis) was equilibrated at 50 °C for 1 min, heated to 95 °C at 12 °C/min, maintained at 95 °C for 2.5 min, cooled to 50 °C at 12 °C/min, and then maintained at 50 °C for 1 min. The paddle speed was set at 960 rpm for the first 10 s, and then decreased to 160 rpm for the rest of the analysis.

A differential scanning calorimeter (DSC 200 F3 Maia) from NETZSCH (Germany) was used to determine the thermal properties of the flour. Each sample (5 mg, dry weight) was loaded into an aluminum pan (25/40 μL, D = 5 mm) and distilled water was added to achieve a flour–water suspension containing 66.7% water. Samples were hermetically sealed and allowed to stand for 24 h at 48 °C before heating in the DSC. The DSC analyzer was calibrated using an empty aluminum pan as a reference. Sample pans were heated at a rate of 10 °C/min from 20 to 100 °C. Thermal transitions of flour samples were defined as onset temperature (*T*_o_), peak of gelatinization temperature (*T*_p_), conclusion temperature (*T*_c_) and the enthalpy of gelatinization (Δ*H*_gel_). After conducting thermal analysis, the samples were stored at 4 °C for 7 days for retrogradation studies. The sample pans containing the starches were reheated at the rate of 10 °C /min from 20 to 100 °C after 7 days to measure retrogradation. The enthalpies of retrogradation (Δ*H*_ret_) were evaluated automatically and the retrogradation percentage (%*R*) was calculated as Δ*H*_ret_/Δ*H*_gel_ × 100.

### 4.7. Statistical Analysis

Data were analyzed for analysis of variance (ANOVA) with SPSS (version 25, IBM, Armonk, NY, USA). The data were homogeneity-tested prior to ANOVA. The homogeneity of the outputs was satisfied for running further ANOVA. Differences in grain yield and quality, as affected by N fertilizer, K fertilizer, year and their interactions, were examined using a three-factor model of ANOVA. The three-factor variance model of ANOVA was used in Supplementary Materials to analyze the effects of year, variety, fertilization and their interactions on grain yield and quality. The differences between the mean values were compared using Duncan’s multiple range test at a *p* < 0.05 level of significance. The relative dependence was determined by the method of correlation analysis (Pearson correlation coefficients), and the obtained coefficients were tested by t-test for significance levels of 0.05% and 0.01%. Figures were generated with OriginPro (version 2022, OriginLab, Northampton, MA, USA).

## 5. Conclusions

Appropriate application of N and K fertilizers could improve the yield and its components of waxy maize, 75 kg ha^−1^ K_2_O at sowing date and top-dressed 75 kg ha^−1^ K_2_O at V6 could not only effectively improve the pasting and thermal properties of waxy maize flour, but also stabilize the grain yield under the conditions of appropriate N reduction. Therefore, we recommend optimized K application (75 kg ha^−1^ at both sowing date and V6) and moderately reduced N application (from 225 to 180 kg ha^−1^) in the production of spring-sown waxy maize in Southern China.

## Figures and Tables

**Table 1:** Interactive effects of nitrogen and potassium on grain yield and yield components of waxy maize.

**Year**	**Fertilization**	**SYN5**				**SYN11**		
		**Kernels per**	**1000-Kernel**	**Grain Yield**		**Kernels per**	**1000-Kernel**	**Grain Yield**
		**Ear**	**Weight (g)**	**(kg ha^−1^)**		**Ear**	**Weight (g)**	**(kg ha^−1^)**
2020	N180K0	415.7c	261.5f	7584.1e		394.2e	296.3e	8148.1d
	N180K75	462.2a	266.3ef	8587.6c		416.8d	315.0ab	9159.0c
	N180K150	463.2a	271.0de	8757.3c		449.4ab	301.4de	9450.8b
	N180K75 + 75	441.2b	284.9c	8769.4c		443.3bc	304.5cd	9414.3b
	N225K0	410.8c	275.8d	7900.8d		389.1e	302.8d	8218.4d
	N225K75	459.4a	286.1bc	9170.4b		448.8ab	309.7bc	9696.4a
	N225K150	455.5a	291.9b	9275.4ab		437.9c	317.7a	9705.7a
	N225K75 + 75	431.2b	313.2a	9423.4a		458.5a	310.4b	9928.8a
2021	N180K0	497.1d	247.1e	8570.9f		483.4d	280.8de	9468.3f
	N180K75	520.3b	253.3cd	9193.9d		498.0c	280.0de	9727.9de
	N180K150	541.6a	250.7de	9472.7bc		485.3cd	294.1c	9955.1cd
	N180K75 + 75	523.2b	261.8b	9555.0b		548.0a	275.8e	10540.9ab
	N225K0	485.5e	264.2b	8950.2e		481.4d	284.6d	9556.0ef
	N225K75	506.9c	264.9b	9368.6c		516.7b	279.7de	10081.6c
	N225K150	535.7a	256.2c	9575.6b		483.8d	310.2a	10468.2b
	N225K75 + 75	508.5c	275.2a	9761.0a		511.3b	301.0b	10738.8a
*F-value*								
N		29.7 **	329.5 **	179.4 **		0.3	69.7 **	68.0 **
K		148.4 **	107.5 **	347.5 **		126.2 **	35.9 **	217.6 **
Y		1965.4 **	602.8 **	516.6 **		1328.2 **	345.0 **	493.7 **
N × K		0.5	3.2 *	0.9		17.6 **	19.9 **	4.5 *
N × Y		2.4	24.4 **	30.4 **		11.1 **	6.9 *	0.5
K × Y		16.2 **	22.0 **	24.7 **		29.9 **	25.7 **	24.3 **
N × K × Y		0.6	5.5 **	5.0 **		12.5 **	5.8 **	2.7

N180, N225: application 180 and 225 kg N ha^−1^; K0, K75, K150: application 0, 75 and 150 kg K_2_O ha^−1^ at sowing date; K75 + 75: application 75 kg K_2_O ha^−1^ at sowing date and jointing stage (V6), respectively. Values followed by a different small letter within a column were significantly different at the 0.05 probability level. * and ** represent significance at the 0.05 and 0.01 probability levels.

**Table 2:** Interactive effects of nitrogen and potassium on grain components of waxy maize.

**Year**	**Treatment**	**SYN5**				**SYN11**		
		**Starch**	**Soluble Sugar**	**Protein**		**Starch**	**Soluble Sugar**	**Protein**
		**(mg g^−1^)**	**(mg g^−1^)**	**(mg g^−1^)**		**(mg g^−1^)**	**(mg g^−1^)**	**(mg g^−1^)**
2020	N180K0	624.1e	36.2cd	90.9bcd		556.8f	32.8c	96.3c
	N180K75	701.5a	32.4e	89.9cd		656.2b	32.9c	100.4a
	N180K150	625.0e	35.5d	82.7e		589.3e	31.3d	98.5b
	N180K75 + 75	634.2d	37.3bc	86.8de		619.7d	33.0c	90.6f
	N225K0	686.0b	40.5a	91.4bc		647.9c	37.6a	94.6d
	N225K75	653.2c	35.6d	95.2ab		596.3e	36.0b	99.9ab
	N225K150	650.5c	38.2b	98.6a		626.3d	33.3c	92.6e
	N225K75 + 75	705.5a	41.1a	92.5bc		673.4a	38.1a	93.0de
2021	N180K0	659.9d	34.9c	89.9d		573.4f	37.1c	87.4de
	N180K75	680.4b	35.8b	94.9a		648.1c	34.7e	90.2bc
	N180K150	669.0c	39.2a	92.8c		577.9f	37.3c	93.5a
	N180K75 + 75	663.1cd	38.9a	93.9b		685.8b	32.3f	86.1de
	N225K0	679.4b	31.5e	83.2g		637.3d	40.6a	90.8b
	N225K75	660.9d	30.3f	88.7e		634.4d	39.3b	85.2e
	N225K150	645.8e	31.1ef	92.2c		626.5e	35.9d	95.4a
	N225K75 + 75	699.1a	34.0d	88.0f		700.7a	30.3g	88.1cd
*F-value*								
N		140.7 **	25.1 **	4.0		583.7 **	532.3 **	1.6
K		98.7 **	83.0 **	8.5 **		650.8 **	218.5 **	57.3 **
Y		55.7 **	189.2 **	1.2		147.0 **	214.4 **	372.0 **
N × K		233.5 **	11.5 **	21.3 **		386.9 **	73.5 **	13.5 **
N × Y		88.2 **	544.0 **	135.0 **		0.8	147.0 **	10.1 **
K × Y		19.4 **	22.7 **	8.5 **		88.2 **	343.7 **	55.4 **
N × K × Y		47.5 **	3.0 *	3.6 *		63.0 **	73.5 **	18.7 **

N180, N225: application 180 and 225 kg N ha^−1^; K0, K75, K150: application 0, 75 and 150 kg K_2_O ha^−1^ at sowing date; K75 + 75: application 75 kg K_2_O ha^−1^ at sowing date and jointing stage (V6), respectively. Values followed by a different small letter within a column were significantly different at the 0.05 probability level. * and ** represent significance at the 0.05 and 0.01 probability levels.

**Table 3:** Interactive effects of nitrogen and potassium on thermal properties of waxy maize.

**Year**	**Treatment**	**SYN5**							**SYN11**						
		**Δ*H*_gel_** **(J g^−1^)**	***T*_o_** **(℃)**	***T*_p_** **(℃)**	***T*_c_** **(℃)**	**Δ*H*_ret_** **(J g^−1^)**	**%*R*** **(%)**		**Δ*H*_gel_** **(J g^−1^)**	***T*_o_** **(℃)**	***T*_p_** **(℃)**	***T*_c_** **(℃)**		**Δ*H*_ret_** **(J g^−1^)**	**%*R*** **(%)**
2020	N180K0	8.7a	71.2a	76.9ab	83.6ab	3.2c	36.5bc		8.0ab	70.3b	75.9bc	82.7b		3.2bc	39.7a
	N180K75	8.3a	71.2a	76.5c	82.8bc	4.3a	51.8a		7.1ab	70.9a	76.2ab	82.4b		3.4abc	47.4a
	N180K150	8.9a	71.4a	76.6bc	83.2bc	4.1ab	45.6ab		8.3a	70.4b	76.0abc	82.4b		3.6ab	44.1a
	N180K75+75	7.2b	71.5a	77.2a	83.5bc	3.3bc	46.1ab		7.4ab	69.8c	75.3d	82.0b		3.4ab	46.7a
	N225K0	9.0a	70.5b	76.0d	82.6c	3.6abc	40.3bc		8.0ab	70.3b	75.8c	82.9b		3.6ab	44.6a
	N225K75	8.6a	71.2a	76.4c	82.6bc	3.3bc	38.7bc		8.2a	70.8a	76.2a	83.8a		4.1a	50.4a
	N225K150	9.1a	71.5a	77.1a	84.4a	3.2c	35.0c		6.9b	70.2b	75.8c	82.5b		2.7c	40.1a
	N225K75 + 75	8.7a	71.4a	76.7bc	82.9bc	3.9abc	45.3ab		7.8ab	70.1bc	75.4d	82.2b		3.7ab	46.6a
2021	N180K0	9.0ab	72.3ab	77.6ab	82.8a	3.0c	33.7bc		8.3abc	70.7b	76.3bc	83.2abc		3.2bc	38.5abc
	N180K75	7.8b	72.3abc	77.5bc	83.2a	3.1c	39.6abc		8.9a	70.8b	76.3bc	83.4a		3.4ab	38.9abc
	N180K150	8.1b	72.1abc	77.4bcd	83.3a	3.1c	38.4abc		7.8cd	70.8b	76.3bc	82.8cd		2.7c	35.3c
	N180K75 + 75	7.8b	72.5a	77.6ab	83.1a	3.2bc	41.6ab		8.6ab	70.9b	76.2c	83.3ab		3.8a	44.7a
	N225K0	10.0a	71.8c	77.2de	83.2a	3.7ab	37.2abc		7.6d	71.3a	76.4b	83.0bcd		3.3b	42.8ab
	N225K75	10.0a	72.0bc	77.3cde	83.3a	3.3bc	33.1c		8.2bc	70.8b	76.1d	82.8cd		3.0bc	36.9bc
	N225K150	8.4b	72.0bc	77.8a	83.2a	3.0c	36.4bc		7.8cd	71.4a	76.6a	82.9cd		3.1bc	39.1abc
	N225K75 + 75	9.1ab	72.0bc	77.2e	83.6a	4.0a	44.7a		8.7ab	70.7b	76.1d	82.7d		3.0bc	35.1c
*F-value*															
N		22.3 **	15.5 **	20.0 **	0.1	1.2	4.5 *		0.2	4.8 *	0.3	1.1	0.9		0.7
K		4.2 *	5.1 **	12.8 **	2.9 *	1.5	5.8 **		7.4 **	12.3 **	21.2 **	8.2 **	8.8 **		2.6
Y		0.9	153.2 **	291.4 **	0.1	8.9 **	10.6 **		27.4 **	121.3 **	143.4 **	36.9 **	4.2		30.9 **
N × K		2.3	3.7 *	27.3 **	1.4	8.9 **	5.6 **		2.4	2.0	0.8	0.5	2.5		5.1 **
N × Y		2.8	1.3	1.0	1.6	9.0 **	3.1		8.7 **	6.6 *	0.4	19.8 **	0.9		1.5
K × Y		2.8	3.5 *	8.3 **	2.2	2.4	1.3		4.8 **	14.8 **	17.2 **	5.2 **	0.4		1.8
N × K × Y		1.5	1.1	2.9	4.1 *	1.3	0.5		8.4 **	6.2 **	3.3 *	7.9 **	9.7 **		5.6 **

N180, N225: application 180 and 225 kg N ha^−1^; K0, K75, K150: application 0, 75 and 150 kg K_2_O ha^−1^ at sowing date; K75 + 75: application 75 kg K_2_O ha^−1^ at sowing date and jointing stage (V6), respectively. Δ*H*_gel_: gelatinization enthalpy; *T*_o_: onset temperature; *T*_p_: peak gelatinization temperature; *T*_c_: conclusion temperature; Δ*H*_ret_: retrogradation enthalpy; %*R*: retrogradation percentage. Values followed by a different small letter within a column are significantly different at the 0.05 probability level. * and ** represent significance at the 0.05 and 0.01 probability levels.

## Data Availability

All the data and code used in this study can be requested by email to the corresponding author Guanghao Li at guanghaoli@yzu.edu.cn or Dalei Lu at dllu@yzu.edu.cn.

## Supplementary Materials

The following supporting information can be downloaded at: https://www.mdpi.com/article/10.3390/plants11192528/s1, Table S1. ANOVA for interactive effects of nitrogen and potassium on grain yield and yield components of waxy maize; Table S2. ANOVA for interactive effects of nitrogen and potassium on grain components of waxy maize; Table S3. ANOVA for interactive effects of nitrogen and potassium on starch volume distributions of waxy maize; Table S4. ANOVA for interactive effects of nitrogen and potassium on pasting properties of waxy maize.
