# Supplementary material for: Interactive Effects of Nitrogen and Potassium on Grain Yield and Quality of Waxy Maize
Source: Plants (Basel). 2022 Sep 27;11(19):2528. doi: 10.3390/plants11192528 (PMC9571907; doi:10.3390/plants11192528)
Supplement: Supplementary file 1 [file plants-11-02528-s001.zip › plants-1931748-supplementary.pdf]

Table S1. ANOVA for interactive effects of nitrogen and potassium on grain yield and yield components of waxy maize.

ANOVA		Kernels per ear	1000-kernel weight (g)	Grain yield (kg ha^{-1})
Year (Y)	2020	436.1b	294.3a	8949.4b
	2021	507.9a	273.7b	9686.5a
Variety (V)	SYN5	478.6a	270.3b	8994.8b
	SYN11	465.4b	297.7a	9641.1a
Fertilization (F)	N180K0	447.6e	271.4f	8442.8g
	N180K75	474.3d	278.7e	9167.1e
	N180K150	484.9ab	279.3de	9409.0d
	N180K75+75	488.9a	281.7d	9569.9c
	N225K0	441.7f	281.8d	8656.3f
	N225K75	482.9bc	285.1c	9579.2c
	N225K150	478.2cd	294.0b	9756.2b
	N225K75+75	477.4d	300.0a	9963.0a
F-value				
Y		3180.2**	910.4**	979.2**
V		108.4**	1624.7**	752.8**
F		94.9**	88.4**	254.1**
Y × V		0.2	5.4*	23.7**
Y × F		14.6**	7.2**	23.7**
V × F		33.6**	32.5**	3.0**
Y × V × F		14.9**	22.5**	3.3**

N180, N225: application 180 and 225 kg N ha^{-1}; K0, K75, K150: application 0, 75, 150 kg K_2O ha^{-1} at sowing date; K75+75: application 75 kg K_2O ha^{-1} at sowing date and jointing stage (V6), respectively. Values followed by a different small letter within a column were significantly different at the 0.05 probability level. * and ** represent significant at the 0.05 and 0.01 probability levels.

Table S2. ANOVA for interactive effects of nitrogen and potassium on grain components of waxy maize.

ANOVA		Starch (mg g^{-1})	Soluble sugar (mg g^{-1})	Protein (mg g^{-1})
Year (Y)	2020	640.4b	35.7a	93.4a
	2021	652.6a	35.2b	90.0b
Variety (V)	SYN5	664.8a	35.8a	90.7b
	SYN11	628.1b	35.2b	92.7a
Fertilization (F)	N180K0	603.5g	35.3c	91.1bc
	N180K75	671.6b	33.9e	93.8a
	N180K150	615.3f	35.8b	91.9b
	N180K75+75	650.7d	35.4c	89.3d
	N225K0	662.6c	37.6a	90.0cd
	N225K75	636.2e	35.3c	92.3b
	N225K150	637.3e	34.6d	94.7a
	N225K75+75	694.7a	35.9b	90.4cd
F-value				
Y		1697.5**	31.4**	42.7**
V		188.7**	25.0**	126.6**
F		561.1**	45.5**	19.5**
Y × V		8.1**	365.2**	88.2**
Y × F		106.1**	115.6**	17.1**
V × F		66.1**	148.9**	24.9**
Y × V × F		38.7**	80.1**	29.1**

N180, N225: application 180 and 225 kg N ha^{-1}; K0, K75, K150: application 0, 75, 150 kg K_2O ha^{-1} at sowing date; K75+75: application 75 kg K_2O ha^{-1} at sowing date and jointing stage (V6), respectively. Values followed by a different small letter within a column were significantly different at the 0.05 probability level. * and ** represent significant at the 0.05 and 0.01 probability levels.

Table S3. ANOVA for interactive effects of nitrogen and potassium on starch volume distributions of waxy maize.

ANOVA		Volume percentage (%)			Mean particle size
		<5 μm	5-15 μm	>15 μm	(μm)
Year (Y)	2020	8.03a	34.41a	57.56b	15.78b
	2021	7.99b	30.90b	61.12a	16.11a
Variety (V)	SYN5	8.07a	35.14a	56.80b	15.63b
	SYN11	7.95b	30.17b	61.88a	16.26a
Fertilization (F)	N180K0	8.03bc	35.51a	56.46f	15.57e
	N180K75	7.94e	31.18e	60.88b	16.22a
	N180K150	7.96d	30.72f	61.32a	16.20a
	N180K75+75	8.02b	32.96c	59.02d	15.83c
	N225K0	8.10a	34.50b	57.40e	15.69d
	N225K75	8.02c	32.03d	59.96c	15.99b
	N225K150	8.04b	33.01c	58.94d	15.88c
	N225K75+75	7.95de	31.29e	60.75b	16.18a
F-value					
Y		168.2**	1199.4**	1201.5**	524.8**
V		1267.5**	2405.2**	2462.0**	1883**
F		148.0**	138.8**	142.9**	139.7**
Y × V		88.5**	336.3**	339.8**	307.2**
Y × F		108.0**	18.3**	18.9**	19.6**
V × F		164.8**	70.8**	75.6**	69.7**
Y × V × F		61.2**	62.3**	64.1**	59.2**

N180, N225: application 180 and 225 kg N ha^{-1}; K0, K75, K150: application 0, 75, 150 kg K_2O ha^{-1} at sowing date; K75+75: application 75 kg K_2O ha^{-1} at sowing date and jointing stage (V6), respectively. Values followed by a different small letter within a column were significantly different at the 0.05 probability level. * and ** represent significant at the 0.05 and 0.01 probability levels.

Table S4. ANOVA for interactive effects of nitrogen and potassium on pasting properties of waxy maize.

ANOVA		PV (cP)	TV (cP)	BD (cP)	FV (cP)	SB (cP)	P_{temp} (°C)
Year (Y)	2020	1886.8b	1296.4b	590.4b	1620.4b	324.0a	78.7a
	2021	2241.7a	1352.1a	889.6a	1641.6a	289.5b	77.8b
Variety (V)	SYN5	2056.7a	1334.5a	722.2b	1651.2a	316.7a	78.9a
	SYN11	2071.8a	1314.0b	757.8a	1610.8b	296.9b	77.7b
Fertilization (F)	N180K0	2081.6c	1344.0ab	737.6c	1651.2ab	307.2ab	78.4a
	N180K75	2012.4de	1318.8cd	693.7d	1620.6c	301.8bc	78.5a
	N180K150	1967.5e	1293.0e	674.5d	1598.8c	305.8ab	78.3a
	N180K75+75	2038.2cd	1305.1de	733.1c	1617.4c	312.3ab	78.1a
	N225K0	2198.7a	1360.7a	838.0a	1672.8a	312.1ab	78.0a
	N225K75	2077.2c	1332.3bc	744.9c	1625.7bc	293.4c	78.6a
	N225K150	1996.5de	1305.2de	691.3d	1609.5c	304.3bc	78.4a
	N225K75+75	2142.0b	1335.1bc	806.9b	1652.2ab	317.1a	78.0a
F-value							
Y		1019.4**	95.0**	1976.9**	9.6**	153.0**	51.0**
V		1.8	13.0**	28.0**	35.1**	50.4**	84.0**
F		24.2**	8.0**	36.2**	6.8**	3.4**	1.3
Y × V		10.3**	8.3**	8.2**	15.9**	14.7**	0.4
Y × F		14.2**	9.5**	16.0**	9.1**	4.3**	2.4*
V × F		38.6**	26.0**	38.4**	31.0**	14.4**	1.9
Y × V × F		18.0**	14.8**	19.5**	14.3**	3.3**	2.7*

N180, N225: application 180 and 225 kg N ha^{-1}; K0, K75, K150: application 0, 75, 150 kg K_2O ha^{-1} at sowing date; K75+75: application 75 kg K_2O ha^{-1} at sowing date and jointing stage (V6), respectively. PV: peak viscosity; TV: trough viscosity; BD: breakdown viscosity; FV: final viscosity; SB: setback viscosity; P_{temp}: pasting temperature; cP: centipoise. Values followed by a different small letter within a column are significantly different at the 0.05 probability level. * and ** represent significant at the 0.05 and 0.01 probability levels.
